# The Case for a Smoker's License

**DOI:** 10.1371/journal.pmed.1001342

**Published:** 2012-11-13

**Authors:** Simon Chapman

**Affiliations:** University of Sydney, Sydney, Australia

## Abstract

Tobacco continues to kill millions of people around the world each year and its use is increasing in some countries, which makes the need for new, creative, and radical efforts to achieve the tobacco control endgame vitally important. One such effort is discussed in this *PLOS Medicine* Debate, where Simon Chapman presents his proposal for a “smoker's license” and Jeff Collin argues against. Chapman sets out a case for introducing a smart card license for smokers designed to limit access to tobacco products and encourage cessation. Key elements of the smoker's license include smokers setting daily limits, financial incentives for permanent license surrender, and a test of health risk knowledge for commencing smokers. Collin argues against the proposal, saying that it would shift focus away from the real vector of the epidemic—the tobacco industry—and that by focusing on individuals it would censure victims, increase stigmatization of smokers, and marginalize the poor.

## Abstract 2

In one half of a *PLOS Medicine* Debate, Simon Chapman lays out a case for a smoker's license designed to limit access to tobacco products and encourage cessation.

The prolonged use of tobacco causes the death of about half its users [1], with a billion people this century predicted to die from tobacco caused disease [2]. In particular, the cigarette is an exceptionally dangerous product: no other commodity or human activity causes a remotely comparable number of annual deaths.

The history of tobacco control has seen policies introduced that were initially considered radical, but which rapidly came to be considered normal [3] and essential to the goals of reducing use and the burden of disease caused. No other consumer product is subject to total advertising bans nor required to be sold in plain packaging, as will occur for tobacco in Australia from December 2012 [4]. Again uniquely, 47 nations now require large graphic warnings on tobacco packaging [5]. Smokefree public transport, workplaces, restaurants, bars, and stadiums are common in an increasingly large number of nations. The World Health Organization's Framework Convention on Tobacco Control that requires such measures has been ratified by 176 nations [6].

Despite these developments, tobacco sale is subject to trivial controls compared with other dangerous products that threaten both public and personal safety. Here I describe a proposal for a major development with further potential to reduce tobacco use—the tobacco user's license—and consider several anticipated objections.

## Tobacco Versus Pharmaceuticals Access

Access to firearms, fireworks, explosives, and dangerous chemicals is often heavily restricted for both personal and public safety reasons. However the most instructive comparison with how tobacco products are sold is with the way governments regulate the sale of other drugs: pharmaceuticals. Those substances known to be benign with little potential for harm or that are unlikely to create dependency, tend to be freely available as over-the-counter products in pharmacies, and increasingly in supermarkets and convenience stores. Mild analgesics, cough and cold remedies, and bronchodilators are good examples.

However pharmaceuticals likely to cause health problems if used incorrectly, for too long, or that require users to be monitored so that the drug or dosage can be modified, are sold by pharmacists to patients with prescriptions issued by medical practitioners and increasingly, nurses [7].

### Prescriptions Are “Temporary Licenses”

While prescriptions are strictly speaking a prescriber's note of authority to a pharmacist to dispense restricted drugs to a named individual, the prescription system is in effect a system of temporary licensing to use restricted substances. Travelers carrying restricted drugs across borders can be required to show that they have a “license” to be in possession of some drugs. It is a criminal offence to supply prescription drugs to those without a prescription and those doing so can face pharmacy or medical deregistration, fines, and possibly imprisonment in serious cases.

To obtain their drugs, users must attend a doctor; pay a sometimes significant consultation fee; and if assessed as needing a drug, then visit a pharmacist. There, they will pay again to receive a limited supply of the drug, sometimes with provision for several repeats. After this, users are required to return to a doctor should they need more drugs.

This process is how nearly all nations regulate drugs designed to ease pain, reduce symptoms, prevent disease, and prolong life. It is seen as a sensible, established system designed to prevent misuse of drugs and to better ensure that access to such drugs is supervised in the interest of patient health.

By contrast, tobacco products can be sold by any retailer. Mixed businesses, supermarkets, petrol stations, kiosks, barbers, bars, and vending machines are examples of the nearly ubiquitous tobacco retailing environment [8]. Unlike prescribed pharmaceuticals, smokers can buy unlimited quantities of tobacco. Many nations outlaw sales to minors, but prosecutions are rare and sales to children common. In contrast to the highly regulated way we allow access to life-saving and health-enhancing pharmaceuticals, this is how we regulate access to a product that kills half its long-term users. Prima facie, there would seem to be a case for redressing this bizarre but historically based inconsistency.

## The Tobacco User's License

The proposed smoker's license described below merits serious consideration as a major platform in the tobacco control endgame now being considered in nations with advanced records of reducing smoking. Earlier, less elaborated accounts were described in 2005 [9], and by LeGrande et al. in 2007 [10] and 2009 [11].

### Smart Card Technology

All smokers would be required to obtain a smart swipecard license [12] to transact any purchase from a licensed tobacco retailer. Retailers could not sell to anyone without a card, because precise reconciliation would be required between tobacco stock supplied by wholesalers to retailers and that sold to licensed smokers. Penalties for unreconciled sales to unlicensed persons would be severe, with the threat of the loss of a retail license, as is now the case for pharmacists supplying restricted drugs to anyone without a prescription.

License application could be made on-line or at authorized tobacconists, with supported data-linkable, proof-of-age cross-referencing (passport, driver's license, birth certificate) required to validate identity. The government licensing authority would validate these identities via data linkage then mail the license.

### A Database of All Smokers

With rapidly increasing internet access, most smokers would probably elect to transact their licensing on-line, thereby providing an email address. This information could be used by governments as a way of efficiently communicating new and potentially cessation-motivating information to all smokers, with tailored messages for different age groups. Every time a sale was transacted, data of high specificity would be added to the national database. These data would enable both immediate and longitudinal national, regional, and local monitoring of tobacco sales in ways that could provide invaluable information about smoker responsiveness to tobacco control initiatives as well as industry price discounting and new brand launches. Such information would be of great assistance to policy and program planners wanting to maximize cessation.

### Pre-Commitment to a Maximum Daily Consumption

The smart card license would be encoded with a maximum purchase limit chosen by the licensee at the time of application. There could be three grades of license: one to ten cigarettes per day (maximum 70 per week), 11–20 (maximum 140 per week), and 21–50 (maximum 350 per week). Loose tobacco equivalents could be calculated. A smoker wanting to purchase a pack would request their brand and swipe their license in the smart card terminal. With the speed that credit card and EFTPOS terminals now approve or deny a transaction, the terminal would instantly confirm that the licensee was either able to purchase a new supply or that the chosen limit had been reached, in which case the terminal would display the earliest date when a new supply could be purchased. Limits would be calculated over a 14-day period. Licensed smokers could purchase their chosen quota as infrequently as once every 2 weeks, to avoid the imposition of any need to visit retailers more often.

The more cigarettes a licensee opted for, the higher the fee. Some 90% of smokers regret having started smoking [13] and 40% make a quit attempt each year [14], most failing. Many smokers are known to support tobacco control policies like tax rises and smoking restrictions because they believe such measures will assist them to quit or reduce their consumption [15],[16]. It is likely that some smokers may use the opportunity to set a lower daily limit via a licensing scheme than they might normally smoke in an effort to reduce their usual consumption. Cutting down before quitting is a common approach to eventual cessation [17].

The pre-set daily limit would preclude smokers consuming more than planned unless they borrowed cigarettes from other licensed smokers. As these would be valued by other smokers, such borrowing would be marginal. The limit would also act as a barrier to unplanned “binge” smoking that occurs now, particularly when alcohol is involved [18].

Smokers could also adjust their consumption limit upwards by going on-line and paying the extra licensing fee, in the way consumers are used to doing with changing their internet download limits. At annual license renewal time they could also elect to change their limit.

### Maximum Daily Limit

There would be an upper limit of 50 cigarettes per day, averaged across 14 days. Very few smokers consume more than this. Allowing purchasers to buy more than 50 may encourage some to obtain a license with the intent of on-selling tobacco to unlicensed smokers. A limit of 50 cigarettes is unlikely to attract such enterprise as it would not provide the on-seller with substantial profit.

Failure to purchase one's pre-committed allocation (for example, when travelling overseas or temporarily quitting) would not allow smokers to purchase the backlog of unpurchased supplies at a future date.

### Cost of License Fee

The license fee would neither be trivial nor astronomical. It would be set at a sufficient level to give smokers some pause in deciding whether to obtain or renew their license. Market research could be used to determine the appropriate level. For the sake of illustration, assume that the lowest level (up to ten cigarettes per day) would be US$100 a year (US$0.27 a day) and the highest US$200 (US$0.54 a day); this could be paid in quarterly installments or in full.

### Periodic Renewal

The license would need to be renewed each year. As with initial application, this could be done on-line, as are many annual or periodic payments, or at authorized tobacconists. The status of the renewal would be recognized by smart card terminals in every retail outlet, as would any change in the smoker-determined weekly limit.

### Incentive to Surrender License

There is some evidence that financial reward can stimulate cessation [19]. The incentive to surrender one's license and obtain a cumulative refund of all license fees paid may promote cessation. As a quit incentive, all license fees paid during a smoker's licensed smoking history would be fully refundable, with compound interest. License surrender would be permanent and reapplication not permitted. If a license fee was US$100 for up to ten cigarettes per day, an individual commencing smoking at 18 years of age could collect US$1,000 plus interest if deciding to quit a decade later. Smokers could be reminded of this system via email each year. Consideration should be given to ending this provision in middle age (for example, 40 years of age) as a major incentive to encourage quitting. The 50-year follow-up of the British doctor's cohort study showed that “those who stopped before middle age … had a pattern of survival similar to that of men who had never smoked” [1]. This information could be heavily publicized to promote permanent license surrender at the start of middle age. Those who at the start of the scheme had obtained their first license when aged over 40 could have this extended to 50 years; 50 to 60 years, and so on.

### Cooling-Off Period

Application for license surrender would incorporate a mandatory 6-month “cooling-off” period where smokers could change their mind and cancel their revocation application if they relapsed. Some smokers relapse far beyond 6 months but it may be that ready access to unlimited supplies of cigarettes is an important contributory factor here, and that inability to purchase legally would reduce later relapse. Those who did relapse after license expiry could be encouraged to use nicotine replacement therapy.

### Poor Smokers

As smoking prevalence diminishes, an increasing proportion of smokers are on low incomes and unemployment or disability support. Some individuals in this group may find it hard to pay for a license. This argument has often been used by the tobacco industry to oppose tobacco tax rises. Those groups advocating keeping tobacco tax low perversely seek to “help” poor smokers by keeping tobacco affordable, which encourages use. Poor smokers, as a group, are known to be more responsive to price than those on higher incomes, in terms of both quitting and reducing use [20], so the additional license cost should add to this effect.

### Tourists and Temporary Visitors

Tourists and temporary visitors wanting to purchase tobacco could apply for a license abroad prior to travel, or on arrival in the same way that local cell phones are now hired by travellers during their stay. Provision would exist for licenses of shorter duration, to accommodate short trips. Short-term licence fees would not be refundable.

### Existing, Adult Smokers

The government would announce the scheme a year in advance of its implementation and encourage early application with “early bird” discounts. Consumers are used to this with, for example, the introduction of mandatory highway toll windscreen transponders. Anyone already aged 18 or over who wanted a smoker's license could be thus “grandfathered” and allowed to buy a license if they chose.

### New Smokers to Pass a Test of Risk Knowledge

A person turning 18 who wished to henceforth legally purchase tobacco could apply for a license. However, unlike the commencing cohort of adult smokers at the start of the scheme, newly licensed smokers would have to pass a knowledge of risk test (see examples in Box 1). Applicants for their first driving license must pass knowledge tests. Sometimes these are elementary, but can also involve learning detailed information about braking distances at different speeds and the meaning of a large variety of road signage. To better ensure that new smokers were making an informed choice, something the tobacco industry has long declared that it believes applies to smokers' decisions (“The tobacco industry believes that people who smoke do so fully informed of the reported health risks of smoking”) [21], new applicants would be required to demonstrate a satisfactory level of knowledge that might encompass issues like: (i) probabilities of various diseases in smokers versus non-smokers; (ii) the impact on day-to-day functioning of diseases like emphysema and heart disease; (iii) average number of years lost by continuing smokers; (iv) financial cost of smoking to an individual across increasing durations of smoking; (v) chemical additives used in cigarette manufacture.

**Box 1. Examples of Multiple Choice Questions That Might Be Asked of New Applicants for a Smoking License**

- If 100 people were diagnosed with lung cancer, how many would we expect to be alive in 5 years time?
- What fraction of smokers do you believe will die early because of their smoking?
- On average, how much longer do non-smokers live than people who have smoked for a long time?
- A long-term smoker who dies from a disease caused by his or her smoking can expect to lose how many years off normal life expectancy?
- If a person smokes an average of fewer than 10 cigarettes a day during their lifetime, their chances of dying from a smoking caused disease compared to a 20+ a day smoker are?[list options]:
- How many times would a typical 20 a day smoker inhale smoke deep into their lungs between the ages of 20 and 40?
- If 100 people try to stop smoking, regardless of which method they use, on average how many do you think will not be smoking 12 months later?
- How many known carcinogens (chemicals which are known to cause cancer) are there in cigarette smoke?

Applicants would be given on-line educational material of direct relevance to the test, and a large, growing question bank would be developed on the basis of this material, with random on-screen questions being given to each applicant. Such a test would disadvantage applicants who had intellectual impairment (see below). However, the same concerns apply to any knowledge test, such as for a driving license or requirement to demonstrate understanding of a contract, lease, or other legal transaction.

The tobacco industry might well find the legal implications of such “informed consent to smoke” attractive. Any smoker seeking legal redress later from a tobacco company for having been misled would have passed the test, making such a line of argument difficult to sustain.

### Dysfunctional Smokers

Some young smokers with profound mental health or intellectual disabilities would be unable to pass the licensing test. Such people would be likely to be under care or on a disability pension. Special provision could be made for another adult, carer, or institutional representative to obtain a license on their behalf, after consideration of their circumstances.

### Gradual Increase in the Minimum Age for Purchase

A Singaporean group [22] has proposed that commencing with the birth cohort born in 2000, from the year 2018, anyone turning 18 would be unable to buy tobacco thereafter. The rationale is that current smokers born before 2000 should be the last generation of smokers. However, libertarian objections that adults should be free to take informed risks, as with smoking, may render such a plan politically unacceptable.

However, a possibly less objectionable variation on this idea is that from a given year, the legal age for smoking would be raised each year by 1 year. As very few smokers commence experimenting with smoking after 23 years, the expectation is that the incremental, progressive rise in the legal smoking commencement age would effectively see very few people take up smoking when the minimum legal age reached around 23 years. Some would object that those aged 18 and over are adults who can vote, be conscripted for military service, and so on, and increasing the minimum age for smoking beyond 18 is therefore unreasonable. However, precedents exist for varying age limit restrictions (e.g., legal drinking age of 21 in parts of the United States; legal refusal of car hire to young and very old drivers; age-related insurance premium differences).

## Potential Objections to the Scheme

### We Should Regulate the Industry, Not Smokers

Some may argue that a regulatory strategy focused on smokers rather than on the tobacco industry is inappropriate, and that regulation should be directed “upstream” at the industry and its products. This is a false dichotomy because user licensing is not being proposed as an alternative to industry or product regulation but as complementary to these. A core argument for the licensing of tobacco retailers has always been that removal of the license (and so the right to sell) could be used as a strong deterrent to selling to minors. This has always been a very poorly rated tobacco control strategy because it relies on the direct observation of sales to minors by regulatory agents, and this is often difficult and time-consuming. License cancellations and prosecutions are therefore rare and a so a very weak disincentive to selling to minors. The instant swipecard license verification ensures that retailers only sell to licensed adult smokers. Also, many platforms of industry and product regulation directly affect smokers (price, packaging, pack warnings, duty-free bans, ingredient regulation) so the criticism that an explicitly user-focused form of regulation is somehow problematic seems misplaced.

### Cost of Administration

The costs of the scheme would include administrative staff costs to process license applications, renewals, and license surrender refunds; publicity costs to inform smokers about the scheme; and retail swipecard terminals. The cost of the scheme would be drawn from the licensing fees, with retailers paying all costs associated with the swipecard terminals. Lost cards would incur a replacement charge.

As explained, the accumulated license fees would be in theory all (eventually) refundable to smokers wishing to surrender their licenses. However, not all smokers would surrender their license by the final age limit specified for surrender and refund (40 years). This would leave a large pool of funds that could be used to administer the scheme.

### Further Stigmatization of Smokers?

Every current smoker's experience has been that tobacco products have always been sold alongside other unrestricted commodities. This has powerfully conditioned the view that cigarettes are “ordinary” commodities and that a proposal like this is self-evidently draconian. Some smokers may feel that they are being treated like registered addicts, and that the license epitomizes their stigmatization [23],[24].

Such understandable reactions reflect many decades of smoking being considered “normal.” Open sale of tobacco is consonant with the lack of understanding of tobacco's harmfulness when cigarettes became a mass distributed and advertised commodity at the start of the twentieth century. However, today's smokers have all experienced a range of profound changes in the way that smoking and cigarettes are socially perceived and regulated. Having to go outside to smoke in now virtually any indoor public setting, having disturbing graphic warnings on packs, and regular exposure to public awareness campaigns resting on negative sub-texts about the undesirability of smoking have all coalesced to drive smoking lower and to stimulate most smokers make quit attempts and regret having started. It would be almost unimaginable for a smoker today to express the hope that their own children would grow up to smoke as well.

The requirement to have a prescription (a temporary license) to legally obtain pharmaceuticals is never decried as stigmatizing or insulting. Those responsible for planning the introduction of smokers' licenses could try to amplify this analogy.

### Licensing Is Unprecedented and Would “Sanction” Smoking

Many nations register methadone users and some allow registered heroin-dependent people access to heroin (Switzerland, Netherlands). In California, Canada, and The Netherlands, licenses are issued for the medicinal use of cannabis. The Northern Territory government in Australia has introduced a photo-ID system integrated with limits on the purchasing of bulk, cheap wine and large single purchase amounts of alcohol [25]. In Australia, the over-the-counter purchase of cold-relief medicines containing pseudoephedrine involves one's identification and address being recorded in a national database, as a means to limit supply to reduce diversion into illicit methamphetamine manufacture [26]. In all of these examples, different forms and levels of drug-user licensing have been introduced as a means of both allowing limited access to different drugs while controlling wider use. Tobacco, which harms far more people than all those drugs combined, currently has no form of user regulation. (In Japan where cigarette sales are dominated by vending, smokers wanting to use the machines must have licenses [27], but the system is incomparable to the current proposal in every other respect.)

### A Slippery Slope?

Opponents of the idea would be quick to suggest that Orwellian social engineers would soon be calling for licenses to drink alcohol and to eat junk food or engage in any “risky” activity. This argument rests on poor public understanding of the magnitude of the risks of smoking relative to other cumulative everyday risks to health. Other than religious-based restrictions on alcohol sales in some Islamic nations, no other product is subject to the restrictions routinely applied to tobacco marketing and packaging in many nations today. In Australia, the first restrictions on tobacco advertising commenced in 1976—36 years ago. Since then, similar restrictions have not been implemented for any other consumer good. Any slope would appear to be decidedly unslippery.

### Black Market Concerns

Might licensing cause a growth in black market tobacco? As obtaining a license would be neither onerous nor very expensive (relative to the cost of smoking itself), there would be few reasons why most current smokers intending to continue would not obtain one. A license would enable easy access to tobacco purchasing, whereas those without a license would need to take trouble to find illicit sources of supply. Here, some would argue that illicit drug trade flourishes in some nations in spite of such drugs needing to be sourced illegally from criminals. The implication here is that many smokers would be similarly willing to transact with criminals. However, this analogy is badly flawed because while illicit drugs can only be sourced illegally, tobacco would still be readily obtainable legally. It is therefore difficult to foresee why significant proportions of smokers would elect to source their tobacco “underground,” dealing with criminals simply because of an easily obtained licensing requirement.

The main explanations for high demand for illicit tobacco are the cheaper price at which illicit tobacco sells, the ease of cross border traffic in some nations, and the high levels of corruption in which illicit trade can flourish [28]. None of these factors would in any way be influenced by a user-licensing system and so are not arguments against licensing.

## Conclusion

The current suite of comprehensive tobacco control policies, embodied in the Framework Convention on Tobacco Control [6], was developed during decades when sometimes large majorities of populations smoked (particularly males). Today, nations that have taken tobacco control seriously have smoking prevalence near or below 20% and are setting medium-term prevalence targets of 10%. Discussions about “endgame” strategy are becoming more common in tobacco control circles [29] and have begun to be articulated by governments and the public: New Zealand has announced a goal of being smokefree by 2025 [30]. In England, 45% of the population and one-third of smokers support a total ban on the sale of tobacco products [31].

In the past 30 years, many nations have introduced legislation for tobacco control that previously seemed unimaginable: total and sponsorship advertising bans, widespread smokefree policies, large graphic warnings, and now, plain packaging. A smoker's license may today seem a radical step toward ending the epidemic of tobacco cause disease, but it is far less radical than prohibiting the sale of tobacco, which is not a strategy that has yet been supported by any international expert report or political forum. The New Zealand government, in setting its 2025 “smokefree” goal, has not said it would actually prohibit the sale of tobacco. A smoker's license allows smokers the choice to continue smoking within a regulatory framework that promises new disincentives to smoke and a major financial incentive to quit.

This proposal is unlikely to gain traction in impoverished nations with poor electronic retailing infrastructure, extensive networks of unlicensed tobacco retailers, high corruption indexes, extensive illicit retailing, and where low priority is given to tobacco control. It will be of most interest to high-income nations that are actively pursuing tobacco control goals.

The requirement for a license would send a powerful, symbolic message to all smokers and potential smokers that tobacco was no ordinary commodity, akin to grocery items, confectionary, or any product on unrestricted sale. It would mark tobacco as a product uniquely deserving of such regulation and thereby invite reflection among smokers on why this exceptional policy had been introduced. This step may diminish self-exempting views that smoking is just another, unexceptional risk in “life's jungle” [32].
